# Publication trends in spine research from 2007 to 2016: Comparison of the Orthopaedic Research Society Spine Section and the International Society for the Study of the Lumbar Spine

**DOI:** 10.1002/jsp2.1006

**Published:** 2018-03-23

**Authors:** John T. Martin, Sarah E. Gullbrand, Aaron J. Fields, Devina Purmessur, Ashish D. Diwan, Thomas R. Oxland, Kazuhiro Chiba, Farshid Guilak, Judith A. Hoyland, James C. Iatridis

**Affiliations:** ^1^ Department of Orthopaedic Surgery Duke University Durham North Carolina USA; ^2^ Department of Orthopaedic Surgery University of Pennsylvania Philadelphia Pennsylvania USA; ^3^ Department of Orthopaedic Surgery University of California San Francisco California USA; ^4^ Department of Biomedical Engineering The Ohio State University Columbus Ohio USA; ^5^ Spine Service, St. George and Sutherland Clinical School The University of New South Wales Kogarah Australia; ^6^ Departments of Orthopaedics and Mechanical Engineering The University of British Columbia Vancouver Canada; ^7^ International Collaboration on Repair Discoveries (ICORD) The University of British Columbia Vancouver Canada; ^8^ Department of Orthopaedic Surgery National Defense Medical College Saitama Japan; ^9^ Department of Orthopaedic Surgery Washington University in St. Louis and Shriners Hospitals for Children St. Louis Missouri USA; ^10^ Faculty of Biology, Medicine and Health, Division of Cell Matrix Biology and Regenerative Medicine The University of Manchester Manchester UK; ^11^ Leni and Peter W. May Department of Orthopaedics Icahn School of Medicine at Mount Sinai New York New York USA

**Keywords:** bibliometrics, International Society for the Study of the Lumbar Spine, Orthopaedic Research Society, spine

## Abstract

This study investigated current trends in spine publications of the membership of Orthopaedic Research Society Spine Section (ORS3) and the more global and clinically focused International Society for the Study of the Lumbar Spine (ISSLS). The PubMed database was probed to quantify trends in the overall number of articles published, the number of journals these articles were published in, and the number of active scientists producing new manuscripts. We also evaluated trends in flagship spine journals (*Spine*, *European Spine Journal*, and *The Spine Journal*) and in the *Journal of Orthopaedic Research.* The total number of active ORS3 and ISSLS authors and articles published have increased over the last 10 years. These articles are being published in hundreds of distinct journals; the number of journals is also increasing. Members of both societies published their work in *Spine* more than any other journal. Yet, publications in *Spine* decreased over the last 5 years for both ORS3 and ISSLS members, while those in *European Spine Journal*, and *The Spine Journal* remained unchanged. Furthermore, members of both societies have published in *Journal of Orthopaedic Research* at a consistent level. The increasing number of manuscripts and journals reflects a characteristic intrinsic to science as a whole—the global scientific workforce and output are growing and new journals are being created to accommodate the demand. These data suggest that existing spine journals do not fully serve the diverse publication needs of ORS3 and ISSLS members and highlight an unmet need for consolidating the premiere basic and translational spine research in an open access spine‐specific journal. This analysis was an important part of a decision process by the ORS to introduce *JOR Spine.*

## 1 INTRODUCTION

The Orthopaedic Research Society Spine Section (ORS3) is a subsection of the Orthopaedic Research Society (ORS) focused on spine‐related basic science research. The International Society for the Study of the Lumbar Spine (ISSLS) is a global and clinically focused spine research organization. These groups have similar missions: to advance spine research and patient care through enhanced communication and collaboration across the spine research communities. As the dissemination of research findings ensures forward progress and innovation, current trends in spine publications have been a frequent topic of discussion at the respective meetings of these groups. Discussants questioned how existing journals met the needs of spine scientists, motivating the analysis of the publication trends of the 2 societies. Publication trends in spine‐specific journals have been investigated in previous studies. Sing et al evaluated the prevalence of keywords in abstracts from these journals over 38 years.^1^ Wei et al also focused on spine‐specific journals and evaluated overall publication and citation trends.^2^ In this study, we focus on the publication trends of individual spine researchers, expanding the search beyond spine‐specific journals to identify trends in the “spine field” as a whole using the ORS3 and ISSLS as representative samples from the field. To do so, we systematically probed the PubMed database to identify and compare the publication trends of ORS3 and ISSLS members. We identified articles published over the period from 2007 to 2016 with spine‐related keywords appearing in their titles and quantified trends in the overall number of articles published, the number of journals these articles were published in, and the number of active scientists producing new manuscripts. We also evaluated trends in flagship spine journals (*Spine*, *European Spine Journal*, and *The Spine Journal*) and in the *Journal of Orthopaedic Research.* The data produced by this analysis were an important factor in the decision made by the ORS Board of Directors to introduce *JOR Spine.*

## 2 SEARCH CRITERIA

The PubMed database was surveyed for spine‐related articles published over a 10‐year span (January 1, 2007‐December 31, 2016) by all principal investigators on the ORS3 and ISSLS membership rosters (as of October 2017). First, the self‐reported demographic information from each society's roster was collated to compile the location and discipline (clinician‐scientist vs scientist) of each member. Then, PubMed was searched for publications in which an ORS3 member (that self‐identified as an “Established Investigator”) or an ISSLS member (membership is limited to experienced researchers) appeared as either the first or last author (ie, had a primary role in selecting the journal in which to publish), and if selected spine‐related keywords appeared in the manuscript title (Table 1). Keywords previously identified as the most prevalent in spine‐related publications from 1978 to 2015^1^ were included (that were specific to the spine), as well as additional relevant words/phrases. A MATLAB program (R2016b; MathWorks, Natick, Massachusetts) was used to read the membership rosters and create one PubMed search term for each group that included all the members' initials and last name, designations for first or last author, and links to the spine‐related keywords. Following the search, results were downloaded from PubMed as a text file and read into an EndNote library for formatting (X8; Clarivate Analytics; Philadelphia, Pennsylvania). The author list, title, journal, year, and author address for each publication were exported for analysis in MATLAB. Members with common last names or no middle initial were also matched to their academic address to account for errors. After collecting the list of articles, the journal names from each article were extracted and the impact factors (IFs) of these journals (in 2016) was mined from the 2016 Journal Citation Reports® (Clarivate Analytics, 2017). We also tracked the number of scientists performing spine research by counting the number of “active authors” per year (ie, the number of individual authors that published at least one paper in a given year as first or last author) and used these values to estimate the average productivity of ORS3 and ISSLS authors by year (the number of articles published per author). Independent of our objective to determine publication trends of the ORS3 and ISSLS societies as a whole, the publication record of individual researchers over the 10‐year span was extracted from the data to identify the most productive researchers in the spine community.

**Table 1:** Spine‐related keywords included in the PubMed search terms

spine*	lamina*	stenosis
spinal	tranverse process*	scolio*
intervertebral disc*	spinous process*	sciatica
intervertebral disk*	cervical	spondylitis
motion segment*	thoracic	spondylosis
functional spinal unit*	lumbar	radiculopath*
intradiscal	sacrum	myelopath*
intradiskal	sacral	disc degeneration
nucleus pulposus	sacroiliac joint*	degenerative disc disease*
annulus	coccyx	degenerative disk disease*
anulus	coccygeal	Schmorl*
spinal canal*	ligamentum flavum	Modic
spinal cord*	anterior longitudinal ligament*	discitis
spinal nerve*	posterior longitudinal ligament*	decompression
dorsal root ganglion*	interspinous ligament*	fusion*
dorsal root ganglia*	erector spinae	discectom*
dura mater	multifidus	nucleotom*
cerebrospinal	psoas	laminectom*
notochord*	iliopsoas	laminotom*
facet*	quadratus lumborum	laminoplast*
vertebra*	piriformis	foraminotom*
endplate*	back pain	facetectom*
pedicle*	neck pain	kyphoplast*
foramen	spondylolisthesis	bone morphogenetic protein*
foramina	herniation*	BMP

* Notation indicates the wildcard search feature.

Linear regression was performed to evaluate overall publication trends with time. Publication trends in the flagship spine journals and *Journal of Orthopaedic Research* were analyzed by binning articles into those published from 2007 to 2011 and those published from 2012 to 2016 and compared by the Mann‐Whitney test (*P* < 0.05 considered a significant difference). Results for the number of articles published in individual journals were reported as a percentage of the total ORS3 or ISSLS publications in that year.

## 3 RESULTS

PubMed lists 1107 articles from 143 ORS3 members and 3464 articles from 372 ISSLS members (Table 2). ORS3 members spanned 14 countries but were primarily located in the United States (US‐based members: ORS3 71.3%, ISSLS 31.2%) while ISSLS spanned 29 countries and had primarily a clinical background (clinician‐scientist vs scientist: ORS3 29%/71%, ISSLS 80%/20%). Out of the 75 spine‐related keywords, the top 20 keywords (in terms of the number of hits on PubMed) recovered 89% of the ORS3 publications and 89% of the ISSLS publications.

**Table 2:** Demographics of ORS3 and ISSLS

Total Membership	ORS3	%	ISSLS	%
	143		372	
Overlap	22	15	22	6
*Discipline*				
Clinician‐Scientist	42	29	297	80
Scientist	101	71	75	20
*Country*				
Australia	5	3.5	12	3.2
Austria			1	0.3
Belgium			6	1.6
Brazil	2	1.4	1	0.3
Canada	7	4.9	17	4.6
China	1	0.7	8	2.2
Croatia			1	0.3
Denmark			2	0.5
Egypt			1	0.3
Finland			10	2.7
France			7	1.9
Germany	2	1.4	9	2.4
Greece			3	0.8
Hong Kong	2	1.4	5	1.3
India			4	1.1
Ireland	1	0.7		
Israel			1	0.3
Italy			5	1.3
Japan	9	6.3	73	19.6
Luxembourg			1	0.3
Netherlands	2	1.4	9	2.4
New Zealand			2	0.5
Singapore			4	1.1
South Africa			1	0.3
South Korea	1	0.7	18	4.8
Spain			1	0.3
Sweden			9	2.4
Switzerland	6	4.2	17	4.6
Taiwan	1	0.7		
Turkey			2	0.5
UK	2	1.4	26	7.0
USA	102	71.3	116	31.2

**Figure 1:**
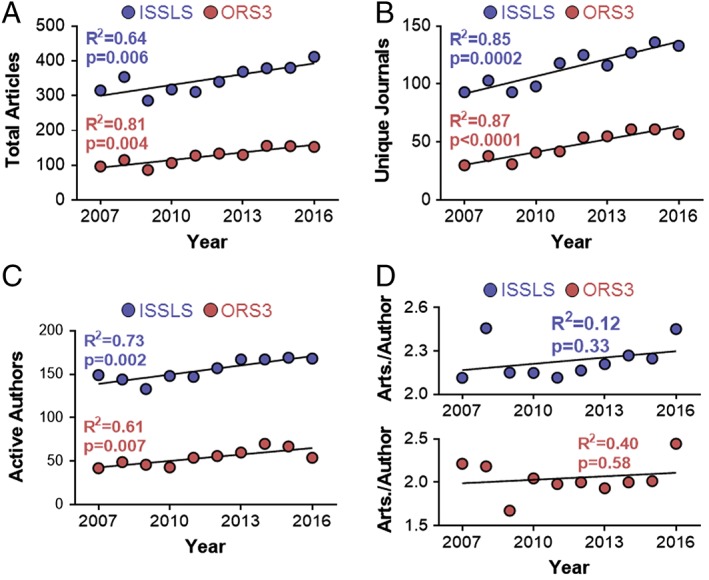
Overall ORS3 and ISSLS member publication trends from 2007 to 2016. A, The total number of articles published by year significantly increased for members of both societies. B, The total number of unique journals that articles were published in by year significantly increased for members of both societies. C, The number of “active authors” (total number of authors that published at least one paper as first or last author in the given year) significantly increased for both societies. D, There was no significant relationship between the number of articles published by active authors and time

**Figure 2:**
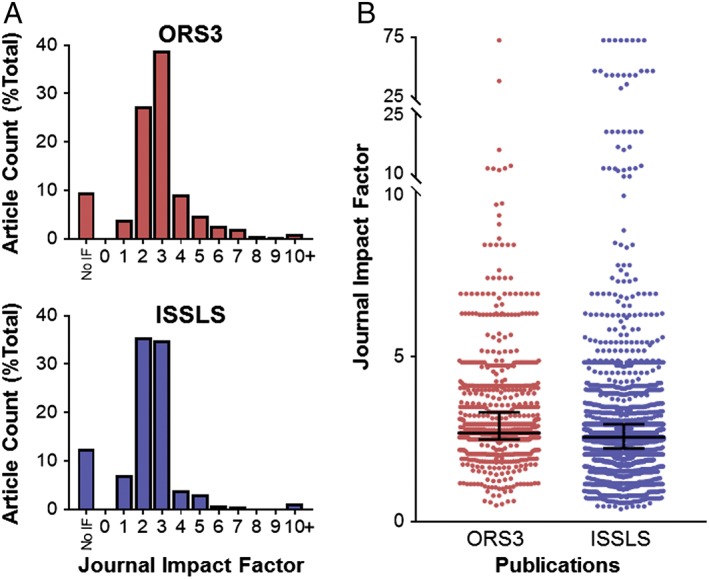
ORS3 and ISSLS publications from 2007 to 2016 sorted by journal impact factor (IF). ORS and ISSLS publications are displayed as a (A) histogram and (B) dot plot (each dot represents an individual publication, with median and interquartile range). Both societies demonstrate a skewed distribution as most articles cluster in the 2 to 3 IF range. ORS3 members publish less articles in lower IF journals and more articles in middle tier journals. ISSLS members published more articles in the highest tier journals

For both groups, the total number of publications and the number of unique journals increased with time (Figure 1). Over the 10‐year span, ORS3/ISSLS members published in hundreds of different journals (OR3 223, ISSLS 494) (Tables [Link], [Link], [Link], [Link]). Similarly, we found that the number of active authors has increased over time (Figure 1C) and that the productivity of these authors has remained relatively constant (Figure 1D). Compared to ISSLS members, ORS3 members published fewer articles in lower IF journals (publications in journals with IF <4: ORS3 73%, ISSLS 80%), and published more articles in middle tier journals (publications in journals with 4 ≤ IF < 10: ORS3 17%, ISSLS 7%) (Figure 2). ISSLS members published more articles in the highest tier journals (publications in journals with IF >10: ORS3 0.7%, ISSLS 1.2%; ie, *New England Journal of Medicine*, *Lancet*, *Journal of the American Medical Association*, *Science*). Many manuscripts (ORS3 35%, ISSLS 28%) went to journals that published less than 10 ORS3/ISSLS member publications over the 10‐year span (journals with <10 member publications over the 10‐year span: ORS3 91%, ISSLS 90%). Furthermore, many manuscripts (ORS3 10%, ISSLS 13%) were published in journals that did not have an IF as they were either less than 2 years old or did not meet quality criteria for the Journal Citation Reports® (journals with no IF: ORS3 19%, ISSLS 22%). There were 20 scientists that had dual ORS3 and ISSLS memberships, accounting for 14% of ORS3 members and 5% of ISSLS members. These members were highly productive and contributed to 29% of the ORS3 publications and 9% of the ISSLS publications. Removing these members from the analysis did not affect the strong correlations between publications and time or unique journals and time.

**Figure 3:**
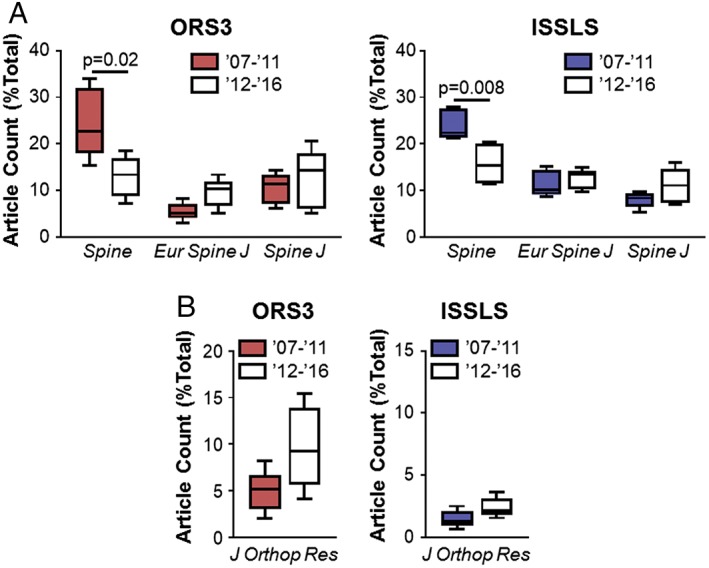
ORS3 and ISSLS member publications in journals of interest in the years 2007 to 2011 and 2012 to 2016. A, Flagship spine journals (*Spine*, *European Spine Journal*, and *The Spine Journal*). There was a significant decrease in the number of articles published in *Spine* for both societies. B, *Journal of Orthopaedic Research.* There were no significant differences in the number of articles published in this journal for either society. Data presented with median and interquartile range

Focusing on individual journals, both the ORS3 and ISSLS groups published most frequently in the flagship spine journals (*Spine*, *European Spine Journal*, and *The Spine Journal*); the top 10 journals ranked by total publication count were dominated by spine‐specific journals (Table 3). In particular, *Spine* was the most prevalent journal for both society members. However, the total ORS3 and ISSLS member publications in *Spine* both significantly decreased from 2007‐2011 to 2012‐2016 (ORS 24.2%‐9.4%, *P* = 0.02; ISSLS 24.1%‐15.6%, *P* = 0.0008) (Figure 3). Publication trends for *European Spine Journal* and *The Spine Journal* remained unchanged. *Journal of Orthopaedic Research* published 6% of ORS3 member articles and 2% of ISSLS member articles from 2007 to 2016 and no significant changes in the number of articles published over time (Figure 3). Of these core journals, there were minor fluctuations in IF over the 10‐year span (range for *Spine*: 2.078‐2.793; *European Spine Journal*: 1.956‐2.563; *The Spine Journal*: 2.426‐3.290; *Journal of Orthopaedic Research*: 2.437‐3.112). Lastly, open access journals emerged in 2012 to 2016 as a frequent publication choice for both societies. From 2007 to 2011, there was one open access journal ranked in the top 10 journals for ORS3 and none for ISSLS; however, there was a shift from 2012 to 2016 as 3 of the top 10 ORS3 journals (*Global Spine Journal*, *PLoS One*, and *Arthritis Research & Therapy*) and 4 of the top 10 ISSLS journals (*Asian Spine Journal*, *Global Spine Journal*, *PLoS One*, and *BMC Musculoskeletal Disorders*) were open access publications.

**Table 3:** ORS3 and ISSLS member publications by journal in the years 2007 to 2011 and 2012 to 2016

ORS3								
	*Years 2007‐2011*				*Years 2012‐2016*			*10 Year Total*
	Total No. of publications	472			Total No. of publications	635		1107
	Total No. of journals	106			Total No. of journals	168		223
*Rank*	*Journal*	*Count*	*%Total*	*Rank*	*Journal*	*Count*	*%Total*	
1	Spine	114	24.2	1	Spine	60	9.4	
2	Spine J	48	10.2	2	Spine J	58	9.1	
3	Eur Spine J	28	5.9	3	J Orthop Res	47	7.4	
4	J Orthop Res	24	5.1	4	Eur Spine J	42	6.6	
5	Arthritis Res Ther^a^	20	4.2	5	J Biomech	36	5.7	
6	J Biomech	16	3.4	6	Global Spine J^a^	22	3.5	
7	J Bone Joint Surg Am	15	3.2	7	J Biomech Eng	14	2.2	
8	J Neurosurg Spine	12	2.5	7	PLoS One^a^	14	2.2	
9	Arthritis Rheum	11	2.3	9	J Neurosurg Spine	11	1.7	
10	Orthop Clin North Am	10	2.1	9	Arthritis Res Ther^a^	11	1.7	

ISSLS								
	*Years 2007‐2011*				*Years 2012‐2016*			*10 Year Total*
	Total No. of Publications	1584			Total No. of Publications	1880		3464
	Total No. of Journals	291			Total No. of Journals	349		494
*Rank*	*Journal*	*Count*	*%Total*	*Rank*	*Journal*	*Count*	*%Total*	
1	Spine	382	24.1	1	Spine	293	15.6	
2	Eur Spine J	181	11.4	2	Eur Spine J	236	12.6	
3	Spine J	127	8.0	3	Spine J	207	11.0	
4	J Neurosurg Spine	45	2.8	4	J Spinal Disord Tech	51	2.7	
5	J Spinal Disord Tech	41	2.6	5	J Orthop Res	46	2.4	
6	J Biomech	28	1.8	6	Asian Spine J^a^	43	2.3	
7	J Bone Joint Surg Am	24	1.5	7	Global Spine J^a^	40	2.1	
7	J Orthop Res	24	1.5	8	J Neurosurg Spine	36	1.9	
9	Clin Biomech	20	1.3	9	PLoS One^a^	27	1.4	
10	J Orthop Sci	15	0.9	10	BMC Musculoskelet Disord^a^	21	1.1	

Journals are ranked in order of the total number of publications over the 5‐year span.

a An open access journal.

Individual authors from the current ORS3 and ISSLS rosters were evaluated for their productivity over the 10‐year span. The most productive authors in the ORS3 were a scientist and a clinician‐scientist who both published 55 articles from 2007 to 2016 (Table S5). In the ISSLS, the most productive author was a clinician‐scientist that published an impressive 122 articles over the same span. In the top 25 most productive ORS3 researchers, there was a 32%/68% clinician‐scientist/scientist split, while for the ISSLS there was an 80%/20% split. This distribution was consistent with the demographics of both societies.

## 4 DISCUSSION

The PubMed database was surveyed for spine‐related research articles published by ORS3 and ISSLS members to characterize the recent publication trends of these societies. The total number of articles published by ORS3 and ISSLS members has significantly increased over the last 10 years, a trend that matches that of the entire spine field^1^, ^2^. These manuscripts were published in hundreds of distinct journals and the total number of journals increased with time. Authors from both ORS3 and ISSLS published most frequently in the flagship spine journals, with *Spine* being the most prevalent. Over the past 10 years, however, there was a significant decrease in the number of publications in *Spine* and this decrease was concomitant with a growing number of journals publishing spine research and the emergence of high quality open access journals (*PLoS One*, *Asian Spine Journal*, *Global Spine Journal*, and *BMC Musculoskeletal Disorders*).

For both societies, only 10% of journals published more than one ORS/ISSLS article per year and the remaining articles were spread across hundreds of journals (Tables S1 and S3). This distribution of articles, with articles concentrated in a core group of journals and the rest spread across a diverse group of journals, is typical of a scientific field and has been documented previously as Bradford's law of scattering,^3^ which describes the distribution of research literature in other medical fields.^4^, ^5^, ^6^, ^7^ The number of distinct journals increased over time (Figure 1B); this may reflect the growing diversity of research interests in the spine field^1^ which is becoming more inter‐ and cross‐disciplinary. Since the number of authors increased with time (Figure 1C) and the number of articles per author remained relatively consistent (Figure 1D), it appears that the total number of articles published is related to the number of active scientists. Overall, the increasing number of authors, manuscripts, and distinct journals in spine research reflects a characteristic intrinsic to science as a whole—the global scientific workforce^8^ and output are growing^9^ and new journals have been created to accommodate the demand.^10^ We believe these results are telling, that the amount of spine research is increasing with time and that there is room for a spine‐focused journal from the established orthopedic research community to support growth in spine research from ORS3, ISSLS, and additional spine societies. We could not, however, track if older researchers exited the academic system between 2007 and 2016, which may affect the overall publication trends. Quantifying “active authors” captures the effects of current younger members ramping up their research programs and current older members slowing theirs, but as the membership rosters were generated in 2016, the analysis does not incorporate older spine researchers that were active in 2007 that retired or slowed their research program. Thus, in these analyses, we used previous findings of growing scientific workforce and output to motivate an assumption that the effects of older members exiting the system were negligible.

The core set of journals for ORS3 and ISSLS were *Spine*, *The Spine Journal*, *European Spine Journal*, and *Journal of Orthopaedic Research. Spine* was the most prevalent journal for members of ORS3 and ISSLS for 2007 to 2011 and 2012 to 2016 (Table 3); however, there was a significant decrease in the number of publications in *Spine* over time (Figure 3A). The decline in *Spine* publications may be related to a reduction in percentage of acceptance and editorial selectivity, the perception of the journal within the spine community, or an increase in the number of journal choices. The second most common journals were *The Spine Journal* for ORS3 members and *European Spine Journal* for ISSLS members (Table 3); this likely reflects the predominantly US‐based ORS3 and more global ISSLS membership. *Journal of Orthopaedic Research* moved up in total publication rank for both ORS3 and ISSLS in the past 5 years (Table 3), suggesting that the broad scientific scope of this journal remains an important venue for publication by ORS3 and ISSLS members. *Arthritis Research & Therapy* and *Arthritis & Rheumatology* (searched as “Arthritis Rheum” and “Arthritis Rheumatol” due to 2014 name change) were ranked in the top 10 ORS3 member periodicals in years 2007 to 2011, however, these journals did not appear on the top 10 list for years 2012 to 2016 (Table 3). The reduction in spine articles in the arthritis journals may reflect changes in the scope of those journals to exclude spine‐related science from the broader arthritis research topics. *Journal of Biomechanics* and *Journal of Biomechanical Engineering* figure prominently in the top 10 journal choices of ORS3 members suggesting that spine biomechanics remains an important research topic area. *Clinical Biomechanics* is no longer an ISSLS top 10 journal choice in the last 5 years (Table 3) which may also suggest changing ISSLS publication needs and/or perceptions of this journal from ISSLS members. Finally, *Journal of Neurosurgery: Spine* was among the top‐ranked journals for ISSLS members (Table 3); this is notable as 77% of the clinician‐scientists in ISSLS list their specialty as orthopedic surgery. The other clinical subspecialties in ISSLS consist of physical medicine (8.5%), neurosurgery (5.9%), rheumatology (2.3%), and physical therapy (2.0%) among others. The publication trends of clinician‐scientists from other specialty societies that also contribute to global spine research, like neurosurgery, would be interesting comparisons beyond the scope of this work.

Two international clinically focused spine journals, *Global Spine Journal* and *Asian Spine Journal*, figure more prominently in the spine research literature over the last 5 years (Table 3). *Asian Spine Journal* “aims to promote communications among spine surgeons especially in Asian countries regarding spine problems and to provide Asian spine surgeons more opportunities to publish their works in international journal” (https://asianspinejournal.org/index.php?body=aims). *Global Spine Journal* is “devoted to the study and treatment of spinal disorders, including diagnosis, operative and non‐operative treatment options, surgical techniques, and emerging research and clinical developments” (https://us.sagepub.com/en-us/nam/global-spine-journal/journal203377#description). These journals may be providing a platform for manuscripts that did not qualify for *Spine* due to an increase in submissions to *Spine.* The relative prominence *Asian Spine Journal* and *Global Spine Journal* (along with *PLoS One*, *BMC Musculoskeletal Disorders*, and *Arthritis Research & Therapy)* also fits an emerging trend of open access publication, which may speak to those journals' ability to understand and meet the needs of modern spine researchers. While basic and translational spine research is accepted at *Asian Spine Journal* and *Global Spine Journal*, their scopes are clinically oriented; there is a potential unmet need for an open‐access journal focused on basic and translational spine research.

There was a significant increase in the number of articles published by ORS3 and ISSLS members reflecting an increase in spine research globally (Figure 1A). In comparing the 2 societies, ORS3 members published more articles in upper‐tier journals (4 < IF < 10), while ISSLS members published more articles in standard journals (IF < 4) and more articles in the highest‐tier journals (IF > 10) (Figure 2, Tables S2 and S4). These findings suggest ORS3 and ISSLS members engage in different avenues of research, and these preferences are likely driven by the demographics of each group; ORS3 is primarily composed of US‐based members that are basic science‐focused, while ISSLS is primarily a globally diverse set of clinician‐scientists.

In conclusion, there are growing numbers of articles published by ORS3 and ISSLS members. The increasing number of publications are distributed in a remarkable number of distinct journals. There has been an increase in prominence of internationally based and open access journals and a decrease in the prominence of *Spine.* These publication trends likely reflect the growing diversity of spine‐related research topics and the increasing globalization of spine research. As a result, we believe that the flagship spine journals do not fully serve the diverse publication needs of ORS3 and ISSLS members. Furthermore, as 90% of journals published less than one ORS/ISSLS article per year, there is an indication that there is no prioritized destination for spine research. These data were used as an important part of a decision process organized by the Orthopaedic Research Society to motivate the introduction of the *JOR Spine.* The success of *JOR Spine* will depend on how it is received by the spine research community. *JOR Spine* is an official publication of the ORS; the support of other international research consortiums has driven the success of the current flagship journals (*Spine*—ISSLS and others, *The Spine Journal*—North American Spine Society, and *European Spine Journal*—EUROSPINE). Furthermore, *JOR Spine* is positioned to account for the demand for globalization, open access publication, and basic science and translational research; to do so, 3 prominent scientists that represent the European, Asian, and North American spine communities will serve as the Editors‐in‐chief of an open access spine‐specific journal. We believe *JOR Spine* will help to consolidate the premiere basic and translational spine science publications that are currently spread among the many journals identified in the analysis above.

## Supporting information


**Table S1** Journals that published from ORS3 members' articles from 2007 to 2016 (sorted by publication count).

**Table S2** Journals that published from ORS3 members' articles from 2007 to 2016 (sorted by impact factor).

**Table S3** Journals that published from ISSLS members' articles from 2007 to 2016 (sorted by publication count).

**Table S4** Journals that published from ISSLS members' articles from 2007 to 2016 (sorted by impact factor).

**Table S5** Most productive individual authors in ORS3 and ISSLS from 2007 to 2016.
