# Supplementary material for: Publication trends in spine research from 2007 to 2016: Comparison of the Orthopaedic Research Society Spine Section and the International Society for the Study of the Lumbar Spine
Source: JOR Spine. 2018 Mar 23;1(1):e1006. doi: 10.1002/jsp2.1006 (PMC5944392; doi:10.1002/jsp2.1006)
Supplement: Supplementary file 1 — Table S1 Journals that published from ORS3 members' articles from 2007 to 2016 (sorted by publication count). [file JSP2-1-e1006-s001.docx]

| **Table S1 - Journals that published from ORS3 members' articles from 2007-2016 (sorted by publication count)** | | | |
| --- | --- | --- | --- |
|  |  |  |  |
| **Rank** | **Journal Name** | **Article Count** | **Impact Factor** |
| 1 | Spine | 174 | 2.499 |
| 2 | Spine J | 106 | 2.962 |
| 3 | J Orthop Res | 71 | 2.692 |
| 4 | Eur Spine J | 70 | 2.563 |
| 5 | J Biomech | 52 | 2.664 |
| 6 | Arthritis Res Ther | 31 | 4.121 |
| 7 | J Biomech Eng | 23 | 2.057 |
| 8 | J Neurosurg Spine | 23 | 2.696 |
| 9 | Global Spine J | 22 | - |
| 10 | J Bone Joint Surg Am | 21 | 4.840 |
| 11 | Eur Cell Mater | 15 | 4.000 |
| 12 | PLoS One | 15 | 2.806 |
| 13 | Tissue Eng Part A | 15 | 3.485 |
| 14 | Arthritis Rheum | 14 | 6.918 |
| 15 | J Spinal Disord Tech | 14 | 2.042 |
| 16 | Acta Biomater | 12 | 6.319 |
| 17 | J Bone Miner Res | 12 | 6.284 |
| 18 | Osteoarthritis Cartilage | 11 | 4.742 |
| 19 | J Biol Chem | 10 | 4.125 |
| 20 | Orthop Clin North Am | 10 | 1.820 |
| 21 | Ann Biomed Eng | 9 | 3.221 |
| 22 | Biomech Model Mechanobiol | 8 | 3.323 |
| 23 | J Am Acad Orthop Surg | 8 | 2.782 |
| 24 | J Anat | 8 | 2.182 |
| 25 | Orthopedics | 8 | 1.143 |
| 26 | J Mech Behav Biomed Mater | 7 | 3.110 |
| 27 | Biomaterials | 6 | 8.402 |
| 28 | Clin Biomech | 6 | 1.874 |
| 29 | Clin Orthop Relat Res | 6 | 3.897 |
| 30 | Comput Methods Biomech Biomed Engin | 6 | 1.909 |
| 31 | Iowa Orthop J | 6 | - |
| 32 | Spine Deform | 6 | - |
| 33 | Biotech Histochem | 5 | 1.041 |
| 34 | Bone | 5 | 4.140 |
| 35 | Curr Stem Cell Res Ther | 5 | - |
| 36 | Exp Mol Pathol | 5 | 2.423 |
| 37 | J Trauma | 5 | - |
| 38 | Matrix Biol | 5 | 7.400 |
| 39 | Am J Pathol | 4 | 4.057 |
| 40 | Biochem Biophys Res Commun | 4 | 2.466 |
| 41 | Connect Tissue Res | 4 | 1.832 |
| 42 | Instr Course Lect | 4 | - |
| 43 | Int J Spine Surg | 4 | - |
| 44 | J Biomed Mater Res A | 4 | 3.076 |
| 45 | J Tissue Eng Regen Med | 4 | 1.169 |
| 46 | Neurosurg Focus | 4 | 3.139 |
| 47 | Neurosurgery | 4 | 4.889 |
| 48 | Oncotarget | 4 | 5.168 |
| 49 | Osteoporos Int | 4 | 3.591 |
| 50 | Scoliosis Spinal Disord | 4 | - |
| 51 | Tissue Eng Part C Methods | 4 | 3.485 |
| 52 | Asian Spine J | 3 | - |
| 53 | BMC Musculoskelet Disord | 3 | 1.739 |
| 54 | Cell Mol Bioeng | 3 | 2.535 |
| 55 | Evid Based Spine Care J | 3 | - |
| 56 | Growth Factors | 3 | 1.644 |
| 57 | JBJS Rev | 3 | - |
| 58 | Mol Genet Metab | 3 | 3.769 |
| 59 | Regen Med | 3 | 2.868 |
| 60 | Sci Rep | 3 | 4.259 |
| 61 | AJNR Am J Neuroradiol | 2 | 3.550 |
| 62 | Am J Sports Med | 2 | 5.673 |
| 63 | Asian J Sports Med | 2 | - |
| 64 | Biores Open Access | 2 | - |
| 65 | Clin Orthop Surg | 2 | - |
| 66 | Clin Spine Surg | 2 | - |
| 67 | Discov Med | 2 | 2.400 |
| 68 | Front Bioeng Biotechnol | 2 | - |
| 69 | Geriatr Orthop Surg Rehabil | 2 | - |
| 70 | Histol Histopathol | 2 | 2.025 |
| 71 | Indian J Orthop | 2 | 0.790 |
| 72 | Int J Mol Sci | 2 | 3.226 |
| 73 | J Biomater Appl | 2 | 2.310 |
| 74 | J Cell Physiol | 2 | 4.080 |
| 75 | J Exp Orthop | 2 | - |
| 76 | J Neurotrauma | 2 | 5.190 |
| 77 | J Orthop Sci | 2 | 1.133 |
| 78 | J Spinal Cord Med | 2 | 1.633 |
| 79 | Macromol Biosci | 2 | 3.238 |
| 80 | Medicine | 2 | 1.804 |
| 81 | Nat Rev Rheumatol | 2 | 12.188 |
| 82 | Orthop Surg | 2 | 1.237 |
| 83 | Orthopade | 2 | 0.629 |
| 84 | Proc Inst Mech Eng H | 2 | 1.005 |
| 85 | Sas j | 2 | - |
| 86 | Stem Cell Res Ther | 2 | 4.211 |
| 87 | Stem Cells | 2 | 5.599 |
| 88 | Stem Cells Int | 2 | 3.540 |
| 89 | Surg Neurol | 2 | 1.669 |
| 90 | World J Orthop | 2 | - |
| 91 | Accid Anal Prev | 1 | 2.685 |
| 92 | Adv Drug Deliv Rev | 1 | 11.764 |
| 93 | Adv Stem Cells | 1 | - |
| 94 | Altex | 1 | 3.825 |
| 95 | Am J Hum Genet | 1 | 9.025 |
| 96 | Am J Physiol Cell Physiol | 1 | 3.602 |
| 97 | Am J Physiol Heart Circ Physiol | 1 | 3.348 |
| 98 | Anat Rec | 1 | 1.431 |
| 99 | Ann Acad Med Singapore | 1 | 0.617 |
| 100 | Ann Am Thorac Soc | 1 | - |
| 101 | Ann Rheum Dis | 1 | 12.811 |
| 102 | Appl Health Econ Health Policy | 1 | - |
| 103 | Autophagy | 1 | 8.593 |
| 104 | BMC Biotechnol | 1 | 2.415 |
| 105 | BMC Genomics | 1 | 3.729 |
| 106 | BMC Health Serv Res | 1 | 1.827 |
| 107 | Biochem Soc Trans | 1 | 2.765 |
| 108 | Biochim Biophys Acta | 1 | 4.702 |
| 109 | Biologicals | 1 | 1.603 |
| 110 | Biomark Insights | 1 | - |
| 111 | Biomed Res Int | 1 | 2.476 |
| 112 | Biorheology | 1 | 1.078 |
| 113 | Biotechnol Bioeng | 1 | 4.481 |
| 114 | Birth Defects Res C Embryo Today | 1 | 3.451 |
| 115 | Bone Joint J | 1 | 2.948 |
| 116 | Bone Joint Res | 1 | 2.597 |
| 117 | Br J Sports Med | 1 | 6.557 |
| 118 | Breast Cancer Res Treat | 1 | 3.626 |
| 119 | Calcif Tissue Int | 1 | 3.124 |
| 120 | Can J Surg | 1 | 1.924 |
| 121 | Cell Dev Biol | 1 | - |
| 122 | Cell Mol Biol | 1 | 0.920 |
| 123 | Cell Tissue Res | 1 | 2.787 |
| 124 | Circ Cardiovasc Interv | 1 | 6.598 |
| 125 | Clin Cancer Res | 1 | 9.619 |
| 126 | Clin J Sport Med | 1 | 2.189 |
| 127 | Clin Sports Med | 1 | 1.475 |
| 128 | Clin Ther | 1 | 2.947 |
| 129 | Cmaj | 1 | 6.784 |
| 130 | Comput Biol Med | 1 | 1.836 |
| 131 | Conf Proc IEEE Eng Med Biol Soc | 1 | - |
| 132 | Congenit Heart Dis | 1 | 1.278 |
| 133 | Crit Rev Eukaryot Gene Expr | 1 | 1.559 |
| 134 | Curr Opin Biotechnol | 1 | 9.294 |
| 135 | Curr Rev Musculoskelet Med | 1 | - |
| 136 | Cytotechnology | 1 | 1.857 |
| 137 | Dev Dyn | 1 | 2.004 |
| 138 | Dev Med Child Neurol | 1 | 3.116 |
| 139 | Dis Model Mech | 1 | 4.691 |
| 140 | Eur J Appl Physiol | 1 | 2.130 |
| 141 | Exp Clin Transplant | 1 | 0.535 |
| 142 | Expert Rev Med Devices | 1 | 2.228 |
| 143 | Faseb j | 1 | 5.498 |
| 144 | Front Surg | 1 | - |
| 145 | Gene Ther | 1 | 3.110 |
| 146 | Genom Data | 1 | - |
| 147 | Histochem Cell Biol | 1 | 2.553 |
| 148 | Hss j | 1 | - |
| 149 | IEEE Trans Biomed Eng | 1 | 3.577 |
| 150 | Immunol Res | 1 | 2.905 |
| 151 | Int J Biol Sci | 1 | 3.873 |
| 152 | Int J Clin Exp Pathol | 1 | 1.706 |
| 153 | Int J Med Sci | 1 | 2.399 |
| 154 | Int J Nanomedicine | 1 | 4.727 |
| 155 | Int J Radiat Oncol Biol Phys | 1 | 5.133 |
| 156 | J Am Coll Surg | 1 | 4.307 |
| 157 | J Appl Biomater Funct Mater | 1 | 1.067 |
| 158 | J Appl Mech | 1 | 2.133 |
| 159 | J Back Musculoskelet Rehabil | 1 | 0.912 |
| 160 | J Biomed Opt | 1 | 2.530 |
| 161 | J Bone Joint Surg Br | 1 | 3.309 |
| 162 | J Cell Biochem | 1 | 3.085 |
| 163 | J Clin Neurosci | 1 | 1.557 |
| 164 | J Dent Res | 1 | 4.755 |
| 165 | J Electromyogr Kinesiol | 1 | 1.510 |
| 166 | J Histochem Cytochem | 1 | 2.511 |
| 167 | J Immigr Minor Health | 1 | 1.314 |
| 168 | J Inflamm | 1 | 2.714 |
| 169 | J Korean Med Sci | 1 | 1.459 |
| 170 | J Mater Sci Mater Med | 1 | 2.325 |
| 171 | J Med Case Rep | 1 | - |
| 172 | J Mol Med | 1 | 4.686 |
| 173 | J Negat Results Biomed | 1 | - |
| 174 | J Neurosci Rural Pract | 1 | - |
| 175 | J Neurosurg Pediatr | 1 | 2.170 |
| 176 | J Neurosurg Sci | 1 | 1.522 |
| 177 | J Oral Maxillofac Surg | 1 | 1.916 |
| 178 | J Orthop Trauma | 1 | 2.251 |
| 179 | J Pain Res | 1 | 2.581 |
| 180 | J Pediatr Orthop | 1 | 1.695 |
| 181 | J Spine Surg | 1 | - |
| 182 | J Steroid Biochem Mol Biol | 1 | 4.561 |
| 183 | J Struct Biol | 1 | 2.767 |
| 184 | J Surg Educ | 1 | 2.163 |
| 185 | J Surg Orthop Adv | 1 | - |
| 186 | J Tissue Eng | 1 | - |
| 187 | J Trauma Acute Care Surg | 1 | 3.403 |
| 188 | J Zoo Wildl Med | 1 | 0.590 |
| 189 | JAMA Intern Med | 1 | 16.538 |
| 190 | Lab Anim | 1 | 1.532 |
| 191 | Lasers Surg Med | 1 | 2.312 |
| 192 | Magma | 1 | 1.718 |
| 193 | Magn Reson Med | 1 | 3.924 |
| 194 | Mech Mater | 1 | 2.651 |
| 195 | Med Biol Eng Comput | 1 | 1.916 |
| 196 | Mol Cell Biomech | 1 | 0.500 |
| 197 | Mol Pain | 1 | 3.533 |
| 198 | N Engl J Med | 1 | 72.406 |
| 199 | NMR Biomed | 1 | 2.872 |
| 200 | Nat Clin Pract Neurol | 1 | 7.636 |
| 201 | Nat Commun | 1 | 12.124 |
| 202 | Nat Mater | 1 | 39.737 |
| 203 | NeuroRehabilitation | 1 | 1.495 |
| 204 | Open Orthop J | 1 | - |
| 205 | Oxid Med Cell Longev | 1 | 4.593 |
| 206 | Pediatrics | 1 | 5.705 |
| 207 | Plast Reconstr Surg | 1 | 3.843 |
| 208 | Pm r | 1 | - |
| 209 | Proc Natl Acad Sci U S A | 1 | 9.661 |
| 210 | Rev Bras Ortop | 1 | - |
| 211 | Rheumatology | 1 | 4.818 |
| 212 | Sao Paulo Med J | 1 | 0.929 |
| 213 | Scoliosis | 1 | - |
| 214 | Skeletal Radiol | 1 | 1.737 |
| 215 | Sports Health | 1 | - |
| 216 | Stapp Car Crash J | 1 | - |
| 217 | Stem Cells Dev | 1 | 3.562 |
| 218 | Surg Neurol Int | 1 | 3.551 |
| 219 | Swiss Med Wkly | 1 | 1.654 |
| 220 | Tissue Eng Part B Rev | 1 | 3.485 |
| 221 | Tohoku J Exp Med | 1 | 1.278 |
| 222 | Trends Dev Biol | 1 | - |
| 223 | World Neurosurg | 1 | 2.592 |
