# Supplementary material for: Publication trends in spine research from 2007 to 2016: Comparison of the Orthopaedic Research Society Spine Section and the International Society for the Study of the Lumbar Spine
Source: JOR Spine. 2018 Mar 23;1(1):e1006. doi: 10.1002/jsp2.1006 (PMC5944392; doi:10.1002/jsp2.1006)
Supplement: Supplementary file 4 — Table S4 Journals that published from ISSLS members' articles from 2007 to 2016 (sorted by impact factor). [file JSP2-1-e1006-s004.docx]

| **Table S4 - Journals that published from ISSLS members' articles from 2007-2016 (sorted by impact factor)** | | | |
| --- | --- | --- | --- |
|  |  |  |  |
| **Rank** | **Journal Name** | **Article**  **Count** | **Impact Factor** |
| 57 | N Engl J Med | 8 | 72.406 |
| 102 | Lancet | 5 | 47.831 |
| 84 | Jama | 6 | 44.405 |
| 466 | Science | 1 | 37.205 |
| 408 | Lancet Oncol | 1 | 33.900 |
| 68 | Bmj | 7 | 20.785 |
| 169 | Ann Intern Med | 2 | 17.202 |
| 348 | JAMA Intern Med | 1 | 16.538 |
| 252 | Ann Rheum Dis | 1 | 12.811 |
| 151 | Nat Rev Rheumatol | 3 | 12.188 |
| 207 | Nat Commun | 2 | 12.124 |
| 237 | Adv Drug Deliv Rev | 1 | 11.764 |
| 264 | Biol Rev Camb Philos Soc | 1 | 11.615 |
| 174 | Brain | 2 | 10.292 |
| 248 | Ann Neurol | 1 | 9.890 |
| 347 | JACC Cardiovasc Interv | 1 | 8.841 |
| 453 | Phys Rev Lett | 1 | 8.462 |
| 173 | Biomaterials | 2 | 8.402 |
| 435 | Neurology | 1 | 8.320 |
| 192 | J Control Release | 2 | 7.786 |
| 436 | Neuro Oncol | 1 | 7.786 |
| 429 | Nat Clin Pract Neurol | 1 | 7.636 |
| 232 | ACS Appl Mater Interfaces | 1 | 7.504 |
| 414 | Matrix Biol | 1 | 7.400 |
| 375 | J Neurol Neurosurg Psychiatry | 1 | 7.349 |
| 157 | Radiology | 3 | 7.296 |
| 67 | Arthritis Rheum | 7 | 6.918 |
| 255 | Arthritis Rheumatol | 1 | 6.918 |
| 387 | J Pathol | 1 | 6.894 |
| 221 | Sports Med | 2 | 6.832 |
| 299 | Cmaj | 1 | 6.784 |
| 427 | Mol Ther | 1 | 6.688 |
| 175 | Br J Sports Med | 2 | 6.557 |
| 165 | Acta Biomater | 2 | 6.319 |
| 73 | J Bone Miner Res | 7 | 6.284 |
| 83 | Cochrane Database Syst Rev | 6 | 6.264 |
| 425 | Mol Neurobiol | 1 | 6.190 |
| 279 | Brain Stimul | 1 | 6.078 |
| 283 | Br J Surg | 1 | 5.899 |
| 430 | Nat Clin Pract Rheumatol | 1 | 5.846 |
| 152 | Neuroimage | 3 | 5.835 |
| 166 | Am J Sports Med | 2 | 5.673 |
| 222 | Stem Cells | 2 | 5.599 |
| 481 | Thyroid | 1 | 5.515 |
| 320 | Faseb j | 1 | 5.498 |
| 282 | Br J Pharmacol | 1 | 5.491 |
| 40 | Pain | 11 | 5.445 |
| 244 | Am J Surg Pathol | 1 | 5.363 |
| 52 | J Neurotrauma | 9 | 5.190 |
| 31 | Neurosurgery | 14 | 4.889 |
| 9 | J Bone Joint Surg Am | 44 | 4.840 |
| 219 | Rheumatology | 2 | 4.818 |
| 59 | Osteoarthritis Cartilage | 8 | 4.742 |
| 251 | Ann N Y Acad Sci | 1 | 4.706 |
| 318 | Exp Neurol | 1 | 4.706 |
| 309 | Dis Model Mech | 1 | 4.691 |
| 402 | J Virol | 1 | 4.663 |
| 446 | Oxid Med Cell Longev | 1 | 4.593 |
| 392 | J Steroid Biochem Mol Biol | 1 | 4.561 |
| 53 | J Pain | 9 | 4.519 |
| 458 | Radiother Oncol | 1 | 4.328 |
| 396 | J Am Coll Surg | 1 | 4.307 |
| 158 | Sci Rep | 3 | 4.259 |
| 489 | Value Health | 1 | 4.235 |
| 125 | Stem Cell Res Ther | 4 | 4.211 |
| 468 | Curr Stem Cell Res Ther | 1 | 4.211 |
| 205 | Med Sci Sports Exerc | 2 | 4.141 |
| 51 | Bone | 9 | 4.140 |
| 187 | J Biol Chem | 2 | 4.125 |
| 226 | J Pediatr | 2 | 4.122 |
| 23 | Arthritis Res Ther | 17 | 4.121 |
| 389 | J Physiother | 1 | 4.083 |
| 116 | J Cell Physiol | 4 | 4.080 |
| 464 | Scand J Work Environ Health | 1 | 4.071 |
| 378 | J Neurosurg | 1 | 4.059 |
| 132 | Ann Surg Oncol | 3 | 4.041 |
| 167 | Anesth Analg | 2 | 4.014 |
| 26 | Eur Cell Mater | 16 | 4.000 |
| 286 | Cancer Sci | 1 | 3.974 |
| 315 | Eur Radiol | 1 | 3.967 |
| 412 | Magn Reson Med | 1 | 3.924 |
| 441 | Occup Environ Med | 1 | 3.912 |
| 17 | Clin Orthop Relat Res | 20 | 3.897 |
| 336 | Int J Biol Sci | 1 | 3.873 |
| 138 | Clin Neurophysiol | 3 | 3.866 |
| 199 | J Sci Med Sport | 2 | 3.857 |
| 215 | Plast Reconstr Surg | 2 | 3.843 |
| 239 | Altex | 1 | 3.825 |
| 358 | Eur J Cardiothorac Surg | 1 | 3.759 |
| 350 | Int J Alzheimers Dis | 1 | 3.731 |
| 303 | Curr Osteoporos Rep | 1 | 3.721 |
| 361 | J Clin Microbiol | 1 | 3.712 |
| 418 | Med J Aust | 1 | 3.675 |
| 310 | DNA Repair | 1 | 3.610 |
| 88 | Osteoporos Int | 6 | 3.591 |
| 108 | Best Pract Res Clin Rheumatol | 4 | 3.581 |
| 469 | Stem Cells Dev | 1 | 3.562 |
| 79 | AJNR Am J Neuroradiol | 6 | 3.550 |
| 317 | Exp Cell Res | 1 | 3.546 |
| 223 | Stem Cells Int | 2 | 3.540 |
| 426 | Mol Pain | 1 | 3.533 |
| 195 | J Neuroeng Rehabil | 2 | 3.516 |
| 459 | Reg Anesth Pain Med | 1 | 3.515 |
| 111 | Clin J Pain | 4 | 3.492 |
| 30 | Tissue Eng Part A | 15 | 3.485 |
| 105 | Tissue Eng Part C Methods | 5 | 3.485 |
| 483 | Tissue Eng Part B Rev | 1 | 3.485 |
| 472 | Aust J Physiother | 1 | 3.481 |
| 50 | Acta Orthop | 9 | 3.446 |
| 147 | J Trauma Acute Care Surg | 3 | 3.403 |
| 374 | J Neurol | 1 | 3.389 |
| 325 | Head Neck | 1 | 3.376 |
| 100 | J Appl Physiol | 5 | 3.351 |
| 220 | Scand J Med Sci Sports | 2 | 3.331 |
| 115 | Joint Bone Spine | 4 | 3.329 |
| 80 | Biomech Model Mechanobiol | 6 | 3.323 |
| 37 | J Bone Joint Surg Br | 12 | 3.309 |
| 42 | Arch Phys Med Rehabil | 10 | 3.289 |
| 438 | Neuroscience | 1 | 3.277 |
| 333 | Integr Biol | 1 | 3.252 |
| 488 | Vaccine | 1 | 3.235 |
| 186 | Int J Mol Sci | 2 | 3.226 |
| 131 | Ann Biomed Eng | 3 | 3.221 |
| 353 | J Biomed Mater Res B Appl Biomater | 1 | 3.189 |
| 268 | Biomol Eng | 1 | 3.172 |
| 103 | Neurosurg Focus | 5 | 3.139 |
| 122 | J Mech Behav Biomed Mater | 4 | 3.110 |
| 266 | Biol Lett | 1 | 3.089 |
| 327 | Health Serv Res | 1 | 3.089 |
| 189 | J Cell Biochem | 2 | 3.085 |
| 118 | J Magn Reson Imaging | 4 | 3.083 |
| 307 | Dig Liver Dis | 1 | 3.061 |
| 96 | Eur J Pain | 5 | 3.019 |
| 156 | Physiotherapy | 3 | 3.010 |
| 293 | Chin J Cancer Res | 1 | 3.000 |
| 196 | J Neurooncol | 2 | 2.980 |
| 3 | Spine J | 334 | 2.962 |
| 81 | Bone Joint J | 6 | 2.948 |
| 181 | Eur J Neurosci | 2 | 2.941 |
| 203 | Med Care | 2 | 2.897 |
| 363 | J Clin Psychopharmacol | 1 | 2.891 |
| 432 | Neuroepidemiology | 1 | 2.886 |
| 60 | Pain Physician | 8 | 2.840 |
| 101 | J Orthop Sports Phys Ther | 5 | 2.825 |
| 36 | Pain Med | 12 | 2.820 |
| 326 | Headache | 1 | 2.816 |
| 15 | PLoS One | 27 | 2.806 |
| 29 | J Am Acad Orthop Surg | 15 | 2.782 |
| 243 | AJR Am J Roentgenol | 1 | 2.778 |
| 262 | Biochem Soc Trans | 1 | 2.765 |
| 61 | Phys Ther | 8 | 2.764 |
| 454 | Phys Med Biol | 1 | 2.742 |
| 367 | J Inflamm | 1 | 2.714 |
| 5 | J Neurosurg Spine | 81 | 2.696 |
| 6 | J Orthop Res | 70 | 2.692 |
| 231 | Accid Anal Prev | 1 | 2.685 |
| 206 | Mol Cell Biochem | 2 | 2.669 |
| 8 | J Biomech | 49 | 2.664 |
| 324 | Gut Liver | 1 | 2.663 |
| 415 | Mech Mater | 1 | 2.651 |
| 86 | Muscle Nerve | 6 | 2.605 |
| 277 | Bone Joint Res | 1 | 2.597 |
| 162 | World Neurosurg | 3 | 2.592 |
| 386 | J Pain Res | 1 | 2.581 |
| 431 | Neurochem Res | 1 | 2.581 |
| 2 | Eur Spine J | 417 | 2.563 |
| 377 | J Neurosci Methods | 1 | 2.554 |
| 242 | Am J Phys Anthropol | 1 | 2.552 |
| 291 | Cell Mol Bioeng | 1 | 2.535 |
| 354 | J Biomed Opt | 1 | 2.530 |
| 14 | Int Orthop | 28 | 2.520 |
| 259 | Asian Pac J Cancer Prev | 1 | 2.514 |
| 1 | Spine | 675 | 2.499 |
| 211 | Pain Pract | 2 | 2.495 |
| 110 | Biomed Res Int | 4 | 2.476 |
| 366 | J Hum Genet | 1 | 2.471 |
| 109 | Biochem Biophys Res Commun | 4 | 2.466 |
| 233 | Acta Anaesthesiol Scand | 1 | 2.438 |
| 451 | Patient Educ Couns | 1 | 2.429 |
| 185 | Int J Colorectal Dis | 2 | 2.426 |
| 97 | Exp Mol Pathol | 5 | 2.423 |
| 271 | BMC Biotechnol | 1 | 2.415 |
| 256 | Artif Organs | 1 | 2.403 |
| 340 | Int J Med Sci | 1 | 2.399 |
| 376 | J Neurophysiol | 1 | 2.396 |
| 372 | J Neural Transm | 1 | 2.392 |
| 261 | Behav Med | 1 | 2.390 |
| 339 | Leg Med | 1 | 2.382 |
| 136 | BMJ Open | 3 | 2.369 |
| 142 | Int J Gynecol Cancer | 3 | 2.369 |
| 302 | Curr Opin Anaesthesiol | 1 | 2.369 |
| 297 | Clin Rheumatol | 1 | 2.365 |
| 416 | Med Decis Making | 1 | 2.362 |
| 98 | Gait Posture | 5 | 2.347 |
| 335 | Int J Immunopathol Pharmacol | 1 | 2.347 |
| 352 | J Athl Train | 1 | 2.341 |
| 368 | J Mater Sci Mater Med | 1 | 2.325 |
| 409 | Lasers Surg Med | 1 | 2.312 |
| 399 | J Neurol Sci | 1 | 2.295 |
| 240 | Am J Med Genet A | 1 | 2.259 |
| 319 | Expert Rev Med Devices | 1 | 2.228 |
| 202 | Magn Reson Imaging | 2 | 2.225 |
| 113 | Hum Factors | 4 | 2.219 |
| 273 | BMC Med Genet | 1 | 2.198 |
| 463 | Scand J Surg | 1 | 2.197 |
| 287 | Cardiovasc Intervent Radiol | 1 | 2.191 |
| 295 | Clin Sports Med | 1 | 2.189 |
| 394 | J Surg Res | 1 | 2.187 |
| 290 | Cell Biochem Funct | 1 | 2.186 |
| 44 | J Anat | 10 | 2.182 |
| 191 | J Clin Monit Comput | 2 | 2.178 |
| 334 | Int Arch Occup Environ Health | 1 | 2.177 |
| 22 | Man Ther | 18 | 2.158 |
| 404 | Korean J Radiol | 1 | 2.156 |
| 296 | Clin Radiol | 1 | 2.141 |
| 208 | Neuroradiology | 2 | 2.093 |
| 155 | Neurosurg Rev | 3 | 2.060 |
| 393 | J Strength Cond Res | 1 | 2.060 |
| 213 | Physiol Meas | 2 | 2.058 |
| 46 | J Biomech Eng | 10 | 2.057 |
| 304 | Curr Pain Headache Rep | 1 | 2.051 |
| 447 | J Paediatr Child Health | 1 | 2.051 |
| 216 | J Plast Reconstr Aesthet Surg | 2 | 2.048 |
| 4 | J Spinal Disord Tech | 92 | 2.042 |
| 449 | Pain Res Manag | 1 | 2.027 |
| 306 | Diagn Pathol | 1 | 2.025 |
| 328 | Histol Histopathol | 1 | 2.025 |
| 300 | Complement Ther Med | 1 | 2.013 |
| 130 | Acta Radiol | 3 | 2.011 |
| 395 | J Am Board Fam Med | 1 | 1.955 |
| 263 | Biol Bull | 1 | 1.950 |
| 92 | Arch Orthop Trauma Surg | 5 | 1.942 |
| 253 | Anticancer Res | 1 | 1.937 |
| 285 | Can J Surg | 1 | 1.924 |
| 314 | Eur J Pediatr | 1 | 1.921 |
| 141 | Exp Brain Res | 3 | 1.917 |
| 123 | Med Biol Eng Comput | 4 | 1.916 |
| 383 | J Oral Maxillofac Surg | 1 | 1.916 |
| 224 | Surg Innov | 2 | 1.909 |
| 301 | Comput Methods Biomech Biomed Engin | 1 | 1.909 |
| 424 | Mol Diagn Ther | 1 | 1.909 |
| 72 | Injury | 7 | 1.894 |
| 305 | Diagn Interv Radiol | 1 | 1.892 |
| 78 | Acta Neurochir | 6 | 1.881 |
| 281 | Br J Biomed Sci | 1 | 1.877 |
| 12 | Clin Biomech | 34 | 1.874 |
| 41 | Spinal Cord | 11 | 1.870 |
| 171 | Appl Ergon | 2 | 1.866 |
| 197 | J Occup Environ Med | 2 | 1.861 |
| 330 | Hum Mov Sci | 1 | 1.841 |
| 139 | Connect Tissue Res | 3 | 1.832 |
| 71 | Eur J Phys Rehabil Med | 7 | 1.827 |
| 272 | BMC Health Serv Res | 1 | 1.827 |
| 474 | J Matern Fetal Neonatal Med | 1 | 1.826 |
| 82 | Clin Anat | 6 | 1.824 |
| 35 | Orthop Clin North Am | 13 | 1.820 |
| 76 | Med Eng Phys | 7 | 1.819 |
| 70 | Ergonomics | 7 | 1.818 |
| 150 | Mod Rheumatol | 3 | 1.818 |
| 69 | Disabil Rehabil | 7 | 1.804 |
| 124 | Medicine | 4 | 1.804 |
| 308 | Disabil Rehabil Assist Technol | 1 | 1.804 |
| 391 | J Sports Sci Med | 1 | 1.797 |
| 390 | J Radiat Res | 1 | 1.788 |
| 38 | Eur Rev Med Pharmacol Sci | 11 | 1.778 |
| 154 | Neurol India | 3 | 1.758 |
| 275 | BMC Vet Res | 1 | 1.750 |
| 11 | BMC Musculoskelet Disord | 36 | 1.739 |
| 344 | Int Neurourol J | 1 | 1.739 |
| 160 | Skeletal Radiol | 3 | 1.737 |
| 66 | Am J Phys Med Rehabil | 7 | 1.734 |
| 236 | Acta Neurol Belg | 1 | 1.722 |
| 410 | Magma | 1 | 1.718 |
| 198 | J Occup Rehabil | 2 | 1.713 |
| 184 | Int J Clin Exp Pathol | 2 | 1.706 |
| 146 | J Pediatr Orthop | 3 | 1.695 |
| 470 | Stereotact Funct Neurosurg | 1 | 1.692 |
| 241 | Am J Med Qual | 1 | 1.688 |
| 85 | J Rehabil Med | 6 | 1.681 |
| 360 | J Clin Anesth | 1 | 1.677 |
| 403 | J Zhejiang Univ Sci B | 1 | 1.676 |
| 126 | Surg Neurol | 4 | 1.669 |
| 225 | Swiss Med Wkly | 2 | 1.654 |
| 323 | Growth Factors | 1 | 1.644 |
| 120 | J Spinal Cord Med | 4 | 1.633 |
| 218 | Res Dev Disabil | 2 | 1.630 |
| 349 | J Altern Complement Med | 1 | 1.622 |
| 193 | J Hand Surg Am | 2 | 1.606 |
| 265 | Biologicals | 1 | 1.603 |
| 493 | World J Surg Oncol | 1 | 1.600 |
| 452 | Phys Med Rehabil Clin N Am | 1 | 1.594 |
| 19 | J Manipulative Physiol Ther | 20 | 1.592 |
| 280 | Braz J Med Biol Res | 1 | 1.578 |
| 74 | J Clin Neurosci | 7 | 1.557 |
| 75 | J Orthop Surg Res | 7 | 1.545 |
| 20 | Yonsei Med J | 20 | 1.537 |
| 407 | Lab Anim | 1 | 1.532 |
| 228 | ScientificWorldJournal | 2 | 1.524 |
| 55 | J Neurosurg Sci | 8 | 1.522 |
| 254 | ANZ J Surg | 1 | 1.513 |
| 18 | J Electromyogr Kinesiol | 20 | 1.510 |
| 345 | Int Urogynecol J Pelvic Floor Dysfunct | 1 | 1.510 |
| 217 | Psychol Health Med | 2 | 1.500 |
| 437 | NeuroRehabilitation | 1 | 1.495 |
| 370 | J Musculoskelet Neuronal Interact | 1 | 1.489 |
| 143 | J Biol Regul Homeost Agents | 3 | 1.469 |
| 58 | Orthop Traumatol Surg Res | 8 | 1.468 |
| 445 | J Orthop Traumatol | 1 | 1.468 |
| 450 | Pathol Int | 1 | 1.465 |
| 117 | J Korean Med Sci | 4 | 1.459 |
| 411 | Magn Reson Imaging Clin N Am | 1 | 1.446 |
| 87 | Neurosurg Clin N Am | 6 | 1.392 |
| 94 | Clin Neurol Neurosurg | 5 | 1.381 |
| 180 | Cleve Clin J Med | 2 | 1.380 |
| 433 | Neurol Res | 1 | 1.376 |
| 170 | Ann R Coll Surg Engl | 2 | 1.350 |
| 465 | Schmerz | 1 | 1.336 |
| 194 | J Int Med Res | 2 | 1.323 |
| 311 | Echocardiography | 1 | 1.314 |
| 460 | Res Vet Sci | 1 | 1.298 |
| 270 | Biosci Biotechnol Biochem | 1 | 1.295 |
| 485 | Traffic Inj Prev | 1 | 1.290 |
| 484 | Tohoku J Exp Med | 1 | 1.278 |
| 316 | Exp Ther Med | 1 | 1.261 |
| 365 | J Eval Clin Pract | 1 | 1.250 |
| 260 | Aust J Prim Health | 1 | 1.246 |
| 444 | Orthop Surg | 1 | 1.237 |
| 482 | Tissue Cell | 1 | 1.232 |
| 245 | Anal Sci | 1 | 1.228 |
| 362 | J Clin Neurophysiol | 1 | 1.224 |
| 229 | Vet Surg | 2 | 1.215 |
| 145 | J Emerg Med | 3 | 1.210 |
| 382 | J Occup Environ Hyg | 1 | 1.200 |
| 238 | Adv Clin Exp Med | 1 | 1.179 |
| 64 | J Tissue Eng Regen Med | 8 | 1.169 |
| 332 | Ind Health | 1 | 1.168 |
| 491 | World J Pediatr | 1 | 1.164 |
| 351 | J Appl Biomater Biomech | 1 | 1.160 |
| 455 | Prim Health Care Res Dev | 1 | 1.147 |
| 32 | Orthopedics | 14 | 1.143 |
| 149 | Minim Invasive Neurosurg | 3 | 1.143 |
| 490 | Vet Radiol Ultrasound | 1 | 1.137 |
| 13 | J Orthop Sci | 31 | 1.133 |
| 467 | Springerplus | 1 | 1.130 |
| 329 | Hong Kong Med J | 1 | 1.111 |
| 357 | J Cardiothorac Surg | 1 | 1.101 |
| 381 | J Obstet Gynaecol Res | 1 | 1.099 |
| 54 | Childs Nerv Syst | 8 | 1.081 |
| 269 | Biorheology | 1 | 1.078 |
| 337 | Int J Clin Exp Med | 1 | 1.069 |
| 417 | Med Hypotheses | 1 | 1.066 |
| 137 | Chin Med J | 3 | 1.064 |
| 93 | Biotech Histochem | 5 | 1.041 |
| 62 | Proc Inst Mech Eng H | 8 | 1.005 |
| 209 | Oper Orthop Traumatol | 2 | 0.977 |
| 172 | Aviat Space Environ Med | 2 | 0.933 |
| 153 | Neurol Med Chir | 3 | 0.929 |
| 292 | Cell Mol Biol | 1 | 0.920 |
| 45 | J Back Musculoskelet Rehabil | 10 | 0.912 |
| 148 | Mil Med | 3 | 0.906 |
| 321 | Forsch Komplementmed | 1 | 0.865 |
| 401 | J Vet Med Sci | 1 | 0.845 |
| 183 | Intern Med | 2 | 0.815 |
| 214 | Physiother Theory Pract | 2 | 0.804 |
| 128 | Acta Med Okayama | 3 | 0.800 |
| 21 | Indian J Orthop | 18 | 0.790 |
| 230 | Work | 2 | 0.779 |
| 177 | Cells Tissues Organs | 2 | 0.776 |
| 428 | Motor Control | 1 | 0.750 |
| 346 | Interv Neuroradiol | 1 | 0.739 |
| 234 | Acta Cir Bras | 1 | 0.729 |
| 373 | J Neurol Surg A Cent Eur Neurosurg | 1 | 0.726 |
| 28 | J Korean Neurosurg Soc | 15 | 0.708 |
| 313 | Eur J Gynaecol Oncol | 1 | 0.692 |
| 434 | Neurologist | 1 | 0.678 |
| 159 | Singapore Med J | 3 | 0.667 |
| 210 | Orthopade | 2 | 0.629 |
| 163 | Z Orthop Unfall | 3 | 0.618 |
| 168 | Ann Acad Med Singapore | 2 | 0.617 |
| 400 | J Pak Med Assoc | 1 | 0.616 |
| 129 | Acta Orthop Traumatol Turc | 3 | 0.599 |
| 65 | Acta Orthop Belg | 7 | 0.576 |
| 486 | Turk Neurosurg | 1 | 0.560 |
| 364 | J Cytol | 1 | 0.476 |
| 342 | Int J Occup Saf Ergon | 1 | 0.469 |
| 106 | Unfallchirurg | 5 | 0.467 |
| 379 | J Nippon Med Sch | 1 | 0.436 |
| 442 | Occup Med | 1 | 0.387 |
| 7 | Asian Spine J | 56 | - |
| 10 | Global Spine J | 41 | - |
| 16 | Scoliosis | 25 | - |
| 24 | Clin Spine Surg | 17 | - |
| 25 | Stud Health Technol Inform | 17 | - |
| 27 | Pm r | 16 | - |
| 33 | Sas j | 14 | - |
| 34 | Eur J Orthop Surg Traumatol | 13 | - |
| 39 | Int J Spine Surg | 11 | - |
| 43 | Instr Course Lect | 10 | - |
| 47 | J Orthop Surg | 10 | - |
| 48 | Rev Med Suisse | 10 | - |
| 49 | Spine Deform | 10 | - |
| 56 | Korean J Spine | 8 | - |
| 63 | Open Orthop J | 8 | - |
| 77 | J Trauma | 7 | - |
| 89 | Rev Bras Ortop | 6 | - |
| 90 | Scoliosis Spinal Disord | 6 | - |
| 91 | Adv Orthop | 5 | - |
| 95 | Clin Orthop Surg | 5 | - |
| 99 | Hss j | 5 | - |
| 104 | Surg Neurol Int | 5 | - |
| 107 | Am J Orthop | 4 | - |
| 112 | Evid Based Spine Care J | 4 | - |
| 114 | JBJS Rev | 4 | - |
| 119 | J Med Case Rep | 4 | - |
| 121 | J Surg Orthop Adv | 4 | - |
| 127 | Ugeskr Laeger | 4 | - |
| 133 | Asian J Endosc Surg | 3 | - |
| 134 | Biores Open Access | 3 | - |
| 135 | BMC Res Notes | 3 | - |
| 140 | Evid Based Med | 3 | - |
| 144 | J Craniovertebr Junction Spine | 3 | - |
| 161 | World J Orthop | 3 | - |
| 164 | Zhonghua Yi Xue Za Zhi | 3 | - |
| 176 | Case Rep Orthop | 2 | - |
| 178 | Zhongguo Xiu Fu Chong Jian Wai Ke Za Zhi | 2 | - |
| 179 | Zhonghua Wai Ke Za Zhi | 2 | - |
| 182 | Fukushima J Med Sci | 2 | - |
| 188 | J Bone Metab | 2 | - |
| 190 | J Child Orthop | 2 | - |
| 200 | J Spine Surg | 2 | - |
| 201 | Lakartidningen | 2 | - |
| 204 | Med Devices | 2 | - |
| 212 | Pain Res Treat | 2 | - |
| 227 | N Z Med J | 2 | - |
| 235 | Acta Med Iran | 1 | - |
| 246 | Anesth Essays Res | 1 | - |
| 247 | Ann Readapt Med Phys | 1 | - |
| 249 | Ann Phys Rehabil Med | 1 | - |
| 250 | Ann Surg Innov Res | 1 | - |
| 257 | Asian J Neurosurg | 1 | - |
| 258 | Asian J Sports Med | 1 | - |
| 267 | Biomark Insights | 1 | - |
| 274 | BMC Sports Sci Med Rehabil | 1 | - |
| 276 | BMJ Case Rep | 1 | - |
| 278 | Brain Nerve | 1 | - |
| 284 | Bull NYU Hosp Jt Dis | 1 | - |
| 288 | Case Rep Surg | 1 | - |
| 289 | Case Rep Vasc Med | 1 | - |
| 294 | Chiropr Man Therap | 1 | - |
| 298 | Clin Pract | 1 | - |
| 312 | Eura Medicophys | 1 | - |
| 322 | Front Bioeng Biotechnol | 1 | - |
| 331 | Conf Proc IEEE Eng Med Biol Soc | 1 | - |
| 338 | Int J Gen Med | 1 | - |
| 341 | Int J Mol Epidemiol Genet | 1 | - |
| 343 | Int J Surg Oncol | 1 | - |
| 355 | J Bodyw Mov Ther | 1 | - |
| 356 | J Brachial Plex Peripher Nerve Inj | 1 | - |
| 359 | J Clin Diagn Res | 1 | - |
| 369 | J Med Biogr | 1 | - |
| 371 | J Negat Results Biomed | 1 | - |
| 380 | J Obstet Gynaecol India | 1 | - |
| 384 | J Orthop Case Rep | 1 | - |
| 385 | J Osteoporos | 1 | - |
| 388 | J Pediatr Neurosci | 1 | - |
| 397 | J Indian Med Assoc | 1 | - |
| 398 | J Indian Soc Pedod Prev Dent | 1 | - |
| 405 | Kyobu Geka | 1 | - |
| 406 | Clin Ter | 1 | - |
| 413 | Manag Care | 1 | - |
| 419 | Med Sci | 1 | - |
| 420 | Methods Mol Biol | 1 | - |
| 421 | Middle East Afr J Ophthalmol | 1 | - |
| 422 | Mo Med | 1 | - |
| 423 | MMW Fortschr Med | 1 | - |
| 439 | Niger Med J | 1 | - |
| 440 | Nihon Naika Gakkai Zasshi | 1 | - |
| 443 | Orthop J Sports Med | 1 | - |
| 448 | Pain Manag | 1 | - |
| 456 | J Prim Health Care | 1 | - |
| 457 | Prof Case Manag | 1 | - |
| 461 | Respir Investig | 1 | - |
| 462 | Saudi J Anaesth | 1 | - |
| 471 | Australas Med J | 1 | - |
| 473 | Iowa Orthop J | 1 | - |
| 475 | J Assoc Physicians India | 1 | - |
| 476 | J Can Chiropr Assoc | 1 | - |
| 477 | Ochsner J | 1 | - |
| 478 | Open Biomed Eng J | 1 | - |
| 479 | Tokai J Exp Clin Med | 1 | - |
| 480 | Gen Thorac Cardiovasc Surg | 1 | - |
| 487 | Turkiye Parazitol Derg | 1 | - |
| 492 | World J Stem Cells | 1 | - |
| 494 | Zhonghua Zhong Liu Za Zhi | 1 | - |
