# Supplementary material for: Publication trends in spine research from 2007 to 2016: Comparison of the Orthopaedic Research Society Spine Section and the International Society for the Study of the Lumbar Spine
Source: JOR Spine. 2018 Mar 23;1(1):e1006. doi: 10.1002/jsp2.1006 (PMC5944392; doi:10.1002/jsp2.1006)
Supplement: Supplementary file 5 — Table S5 Most productive individual authors in ORS3 and ISSLS from 2007 to 2016. [file JSP2-1-e1006-s005.docx]

| **Table 4 - Most productive individual authors in ORS3 and ISSLS from 2007-2016** | | | | | | | | |
| --- | --- | --- | --- | --- | --- | --- | --- | --- |
|  |  |  |  |  |  |  |  |  |
| **ORS3** |  |  |  |  | **ISSLS** |  |  |  |
|  | **Rank** | **Author** | **Count** |  |  | **Rank** | **Author** | **Count** |
|  | *1* | Scientist | 55 |  |  | *1* | Clinician-Scientist | 122 |
|  | *1* | Clinician-Scientist | 55 |  |  | *2* | Clinician-Scientist | 83 |
|  | *3* | Scientist | 48 |  |  | *3* | Clinician-Scientist | 80 |
|  | *3* | Scientist | 48 |  |  | *4* | Clinician-Scientist | 72 |
|  | *5* | Clinician-Scientist | 45 |  |  | *5* | Clinician-Scientist | 65 |
|  | *6* | Scientist | 44 |  |  | *5* | Clinician-Scientist | 65 |
|  | *7* | Scientist | 43 |  |  | *7* | Clinician-Scientist | 61 |
|  | *7* | Clinician-Scientist | 43 |  |  | *8* | Clinician-Scientist | 60 |
|  | *9* | Clinician-Scientist | 35 |  |  | *9* | Clinician-Scientist | 52 |
|  | *10* | Clinician-Scientist | 33 |  |  | *10* | Clinician-Scientist | 50 |
|  | *11* | Scientist | 32 |  |  | *11* | Scientist | 49 |
|  | *12* | Scientist | 28 |  |  | *11* | Clinician-Scientist | 49 |
|  | *13* | Clinician-Scientist | 24 |  |  | *13* | Scientist | 48 |
|  | *14* | Clinician-Scientist | 23 |  |  | *13* | Clinician-Scientist | 48 |
|  | *15* | Scientist | 22 |  |  | *15* | Clinician-Scientist | 46 |
|  | *16* | Scientist | 20 |  |  | *16* | Scientist | 45 |
|  | *16* | Scientist | 20 |  |  | *16* | Clinician-Scientist | 45 |
|  | *18* | Scientist | 18 |  |  | *18* | Scientist | 44 |
|  | *18* | Scientist | 18 |  |  | *19* | Scientist | 43 |
|  | *18* | Scientist | 18 |  |  | *19* | Clinician-Scientist | 43 |
|  | *21* | Clinician-Scientist | 16 |  |  | *19* | Clinician-Scientist | 43 |
|  | *22* | Scientist | 15 |  |  | *19* | Clinician-Scientist | 43 |
|  | *22* | Scientist | 15 |  |  | *23* | Clinician-Scientist | 42 |
|  | *22* | Scientist | 15 |  |  | *23* | Clinician-Scientist | 42 |
|  | *22* | Scientist | 15 |  |  | *25* | Clinician-Scientist | 38 |
